# The Extracellular Matrix: An Accomplice in Gastric Cancer Development and Progression

**DOI:** 10.3390/cells9020394

**Published:** 2020-02-08

**Authors:** Ana Margarida Moreira, Joana Pereira, Soraia Melo, Maria Sofia Fernandes, Patrícia Carneiro, Raquel Seruca, Joana Figueiredo

**Affiliations:** 1 Epithelial Interactions in Cancer Group, i3S-Instituto de Investigação e Inovação em Saúde, Universidade do Porto, 4200-135 Porto, Portugal; amoreira@ipatimup.pt (A.M.M.); jspereira@ipatimup.pt (J.P.); soraiam@ipatimup.pt (S.M.); sfernandes@ipatimup.pt (M.S.F.); pcarneiro@ipatimup.pt (P.C.); rseruca@ipatimup.pt (R.S.); 2 Institute of Molecular Pathology and Immunology of the University of Porto (IPATIMUP), 4200-135 Porto, Portugal; 3 Institute of Biomedical Sciences Abel Salazar (ICBAS), University of Porto, 4050-313 Porto, Portugal; 4 Medical Faculty, University of Porto, 4200-319 Porto, Portugal

**Keywords:** gastric cancer, extracellular matrix, integrin, cell-ECM interaction, ECM deregulation, ECM targeting, ECM biomarkers

## Abstract

The extracellular matrix (ECM) is a dynamic and highly organized tissue structure, providing support and maintaining normal epithelial architecture. In the last decade, increasing evidence has emerged demonstrating that alterations in ECM composition and assembly strongly affect cellular function and behavior. Even though the detailed mechanisms underlying cell-ECM crosstalk are yet to unravel, it is well established that ECM deregulation accompanies the development of many pathological conditions, such as gastric cancer. Notably, gastric cancer remains a worldwide concern, representing the third most frequent cause of cancer-associated deaths. Despite increased surveillance protocols, patients are usually diagnosed at advanced disease stages, urging the identification of novel diagnostic biomarkers and efficient therapeutic strategies. In this review, we provide a comprehensive overview regarding expression patterns of ECM components and cognate receptors described in normal gastric epithelium, pre-malignant lesions, and gastric carcinomas. Important insights are also discussed for the use of ECM-associated molecules as predictive biomarkers of the disease or as potential targets in gastric cancer.

## 1. Introduction

The extracellular matrix (ECM) is a complex assembly of fibrous proteins, proteoglycans and other molecules, namely cytokines, growth factors and hormones, whose precise composition varies from tissue to tissue [1]. A tight ECM regulation provides for proper architecture, as well as essential cues for mechanosensing and signaling during tissue development and maintenance [1,2]. Indeed, cellular processes such as growth, differentiation, survival, and morphogenesis are highly dependent on the cell-ECM interplay [3,4]. The reciprocal relationship between cells and the ECM is mainly mediated by cell receptors for ECM components, the so-called integrins. 

**Figure 1:**
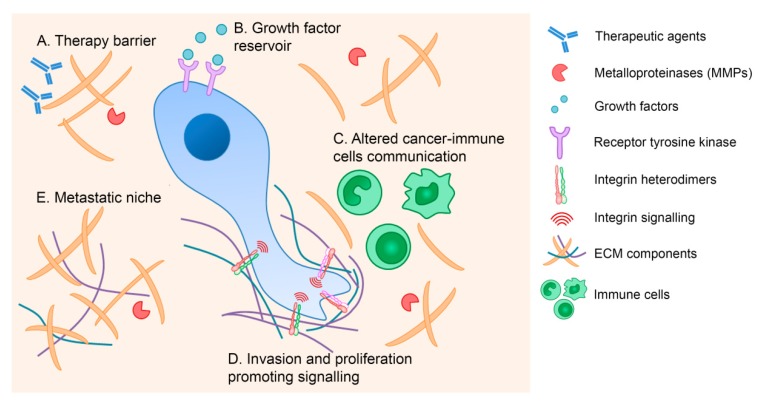
The extracellular matrix (ECM) contribution to cancer pathogenesis. The ECM mediates cancer development through several mechanisms, including formation of a physical barrier to anti-cancer drugs (A), provision of growth factor and cytokines reserves (B), alteration of immune cell responses (C), stimulation of integrin-dependent signaling that promotes invasion and proliferation (D), and establishment of an advantageous niche for metastatic cells (E).

Integrins work as bi-directional molecules that transduce physical and biochemical inputs from the surrounding microenvironment to the cell and vice-versa: transforming intracellular signals into different interactions with their ECM ligands [5,6]. Abnormal ECM and integrin profiles are frequently reported in cancer corroborating the functional relevance and the specificity of both ECM and integrins. Several studies have provided evidence that the ECM contributes to cancer pathogenesis by (i) stimulating integrin-dependent signaling that promotes invasion and proliferation; (ii) promoting an advantageous microenvironmental niche for metastatic cells; (iii) serving as a reservoir of growth factor and cytokines; (iv) interfering with the communication between cancer and immune cells; and (v) forming a physical barrier to anti-cancer agents (Figure 1) [4,6].

In gastric cancer, the role of the ECM has been undeniably demonstrated in all steps of the disease, from initiation to metastases. For instance, increased expression of tenascin has been detected in pre-malignant and malignant gastric epithelia [7], whereas collagens have been shown to be deregulated in more advanced stages [8,9,10]. Notably, a subset of collagen genes have been suggested as powerful independent prognostic markers and are able to distinguish pre-malignant from malignant lesions [8,9,10]. It is thus foreseen that ECM components and interactors hold great clinical potential as prognostic biomarkers and pharmacological targets in gastric cancer.

This review comprises a comprehensive analysis of the main studies concerning ECM remodeling and integrin alterations during gastric cancer development. In particular, we highlight the current understanding of key ECM components and adhesion molecules that mediate aberrant cell-ECM crosstalk and postulate their future application in innovative gastric cancer therapies.

## 2. The Biological Relevance of the ECM in Normal Gastric Tissue

The ECM is a complex three-dimensional network, ubiquitously present in the non-cellular compartment of tissues, providing structural support and maintaining normal tissue architecture, whilst modulating intercellular crosstalk [4,11,12]. As part of every cell’s microenvironment, the ECM is produced and released by local cells such as tissue-specific cells, fibroblasts, and immune cells [1,13,14].

Structurally, the ECM is an organized scaffold assembling a vast number of different macromolecules, namely collagen type-I, -II, -III, -V, and -XI, fibronectin, laminin, vitronectin, elastin, and growth factors, cytokines, and matrix metalloproteinases (MMPs), for which the ECM acts as a reservoir controlling their distribution and availability [12,15]. A complete list of ECM components, their structure, and function are reviewed elsewhere [1,15,16]. Additionally, there are two specialized forms of ECM, the basement membrane, which links the cells to the interstitial matrix, and the interstitial matrix itself, a scaffold in the form of a hydrated gel [11].

It is now well established that the type of components, their proportions, and their assembly determine the rigidity, porosity, and other properties that characterize each tissue [17]. It is thus expected that alterations in ECM composition and assembly, and subsequently on its mechanical and biochemical properties, will strongly affect cellular communication, function, and behavior [4,11,12].

Over the last few years, increasing evidence has emerged on the key role of the ECM in mediating distinct cellular processes including cellular adhesion, polarity, migration, differentiation, proliferation, and survival [14]. Cells are able to sense ECM signals mainly through integrins, a family of cell heterodimeric transmembrane proteins, known to modulate the cytoskeleton and transduce mechanical forces into biochemical events, thus inducing the activation of particular signaling cascades [16,18]. Conversely, cells can also remodel the ECM through the synthesis of new matrix components, alteration of their assembly, or production of enzymes that disrupt the ECM in response to intra- or extracellular stimuli as those occurring during cancer initiation and progression [19,20].

In epithelial tissues, as in gastric epithelia, the basal surface of cells is surrounded by the basement membrane, which is mainly composed of laminins, collagen type-IV, entactin, nidogen, proteoglycans (e.g., perlecan and agrin), and other glycoproteins [11,21,22]. This structure separates the epithelium from the surrounding stroma and provides support and cues for epithelial cell orientation which will help in the establishment and maintenance of the apicobasal polarity [4,23]. While some components are ubiquitously present in the basement membrane scaffold, a wide range of other constituents are unique, depending on the tissue function and origin [24]. In contrast, the interstitial matrix consists mainly of macromolecules such as fibrillar collagens (collagen type-I), fibronectin and proteoglycans, serving a wide variety of purposes, for instance buffering, hydration, binding, and force-resistance of the tissue. [11]. In the human gastric mucosa, aside from the above mentioned common constituents, a specific spatiotemporal distribution of ECM components is known to exist and to differ from that of other epithelial tissues [13]. In the early stages of gastric tissue formation, in which the gastric epithelium is mainly composed of undifferentiated cells, the glycoprotein tenascin was shown to be co-expressed with fibronectin [25]. This expression profile suggests a possible differential role of these ECM components during the morphogenesis of human gastric mucosa [25]. In contrast, other ECM components, for instance, basement membrane heparan sulfate proteoglycan (HSPG) core protein, collagen type-IV, and laminin α1, β1, and γ1 chains were systematically and uniformly detected [25]. More recently, tenascin-X was shown to be critical for normal gastric function as tenascin-X-deficient patients display upper gastric dysfunction and significantly greater symptoms of reflux, indigestion, and abdominal pain [26].

In addition, it was demonstrated that distinct chains of collagen type-IV are differentially distributed in human gastrointestinal tissues [27]. Briefly, collagen, the most abundant ECM fibrous protein, is formed by polypeptide α-chains that assemble into homo- or hetero-trimers and to date up to 28 different types of collagen have been described [28]. Whereas α1(IV), α2(IV), α5(IV), and α6(IV) collagen chains were found in the subepithelial basement membrane of all tissues, the α3(IV) and α4(IV) chains were restricted to specific regions of the gastric and intestinal epithelium, directly facing the lumen of the gastrointestinal tract [27]. This was suggestive of a protective effect against chemical and physical stress, given that α3(IV) and α4(IV) are considered to form physically stronger networks than other collagen type-IV molecules [27,29].

Laminin, another major component of the epithelial basement membrane, is a heterotrimeric protein containing α-, β-, and γ-chains [30]. In human gastric mucosa, laminin chains were shown to have differential expression, with laminin α1 chain found at the basement membrane of both surface and glandular epithelia, while laminin α2 and α3 chains were grossly mutually exclusive. Specifically, α2 chains were mostly detected in the glandular basement membrane and α3 at basement membranes underneath the surface epithelium [31]. The composition of the surface epithelial basement membrane was associated with rapid recovery capacity of the gastric surface epithelium following chemical injury, but also suggested specific programs towards surface or glandular cell differentiation [31].

Another ubiquitous component of the ECM is fibronectin, a dimeric glycoprotein involved in normal cell adhesion and growth, also shown to be central for tissue development and wound healing [32]. Fibronectin modulates the organization of interstitial ECM and is key for cell-ECM interaction as a ligand for many molecules, among which are other ECM components and integrins [11,33]. Supporting its involvement in epithelial healing, fibronectin was found to be markedly increased in the submucosa of healing gastric ulcers when compared to normal controls [34].

Overall, although many aspects of the ECM are still to unravel, it is well established that a tightly controlled ECM composition and cell-ECM interactions are required for normal gastric epithelial function. Deregulation of the ECM will thus result in the development of many pathological conditions, as is the case of gastric cancer.

## 3. ECM Deregulation in Pre-Malignant Lesions and Gastric Cancer

In cancer, transformed cells are able to leave the tissue of origin, invade and home to metastatic niches—a process in which the ECM plays a fundamental role [35]. It is known that, in parallel to alterations in cancer cells, ECM remodeling enzymes (including MMPs) increase their levels; cancer-associated fibroblasts (CAFs), immune cells, and other stromal cells are recruited; various growth factors are secreted, and collagen deposition is induced, all contributing to ECM remodeling at the tumor site [36,37]. These series of events lead to increased ECM stiffness, abnormal cell-cell adhesion, up-regulation of integrin signaling and subsequent activation of downstream cascades, promoting tumor growth and progression [17,36,37].

In addition to recognized cancer-associated ECM alterations, pre-malignant lesions already display ECM deregulation, with major implications for prognosis purposes and therapeutic strategies. In the particular case of human gastric epithelia, it is possible to distinguish two main histological types of gastric adenocarcinoma: the diffuse and the intestinal-type [38]. In the diffuse-type gastric cancer, no pre-malignant lesion is known and, microscopically, it appears to lack glandular structures, consisting of isolated or small groups of poorly cohesive cells [39,40,41]. These cells diffusely infiltrate the gastric wall leading to its widespread thickening and rigidity, known as linitis plastic [42]. In the distinct intestinal-type, a cascade of precancerous lesions—known as the Correa cascade—precedes gastric cancer [43,44]. In this histological type, normal gastric epithelia can evolve into non-atrophic chronic gastritis with active chronic inflammation and advance to multifocal atrophic gastritis, followed by intestinal metaplasia and dysplasia, culminating in gastric adenocarcinoma [43,44]. In the context of pre-malignant lesions and cancer development, tenascin was shown to be differentially expressed in inflammatory, dysplastic and neoplastic lesions of the human stomach (Table 1) [7]. A slight increase of tenascin was observed in superficial inflammation and early cancer, whereas a marked increase was detected in ulcers and invasive tumors of both diffuse and intestinal types, implicating tenascin in malignant growth and lesions undergoing repair and remodeling [7]. Furthermore, a subset of collagen genes was found to be differentially expressed in lesions of the human stomach and able to distinguish malignant from pre-malignant lesions, pinpointing these genes, in particular, COL11A1 and COL1A1, as biomarkers for early detection of gastric cancer [10]. Of relevance, MMP proteins, which belong to a family of zinc-dependent proteolytic enzymes, are important for ECM degradation thus contributing to the disruption of the basement membrane [45]. Accordingly, an increase in MMP-2 and MMP-9 production was observed in the gastric mucosa of patients with *Helicobacter pylori*-associated gastritis when compared with that of uninfected individuals, which indicates that MMP activity is likely to contribute to tissue damage during this process [46]. Subsequent studies have shown that gastric cancer cells infected by *Helicobacter pylori* increase the activity of MMP-2, MMP-9, and MMP-10 through c-Met- and EGFR-dependent signaling pathways, inducing ECM remodeling and cell invasion [47,48].

Further corroborating their involvement in gastric carcinogenesis, altered levels of distinct MMPs have been widely reported in gastric cancer. For instance, MMP-9 expression was markedly higher in gastric carcinoma tissues than in adjacent healthy tissues, and associated with the depth of cancer invasion, suggesting that MMP-9 may serve as a novel biomarker in the diagnosis and prognosis of gastric carcinoma [53]. Moreover, MMP-9 levels were found to be significantly higher in the serum of gastric cancer patients when compared with those of controls [54]. Evaluation of urine samples from individuals with gastric cancer versus healthy controls also revealed that urinary MMP-9/NGAL complex was a potential biomarker of early-stage gastric cancer [57]. In addition, a number of meta-analyses found that overexpression of MMPs was associated with poor prognosis in gastric cancer patients, as was the case for MMP-9 and MMP-2 [50,51,56]. Interestingly, expression levels of both proteins were significantly higher in intestinal-type gastric cancer than in the diffuse-type [49]. MMP-9 expression, along with COX-2 and VEGF, were also increased in gastrointestinal stromal tumors (GIST), the most common mesenchymal neoplasms of the gastrointestinal tract [55]. Likewise, aggressive tumor phenotype and shorter overall survival in gastric cancer patients have been associated with higher MMP-7 expression [52].

Other ECM components are similarly relevant in gastric cancer development, as demonstrated by a large number of studies. Guszczyn and Sobolewski reported the enhancement of collagen turnover in gastric cancer tissues, which could contribute to disorganization of the ECM [59]. Several collagen genes were found to be overexpressed in gastric cancer and, among these, COL1A1 and COL4A1 were closely associated with overall survival of gastric cancer patients and could be regarded as risk factors for poor prognosis [9]. The expression of COL12A1 was also found upregulated in gastric cancer and positively correlated with tumor invasiveness, metastasis, and advanced clinical stage [8]. In tumor tissues of gastric cancer patients, levels of collagen type-I and -IV, fibronectin, and laminin were markedly higher than those detected in the normal tissues [60].

Furthermore, others and our group have demonstrated the importance of laminin γ2, which is a major component of epithelial basement membranes, in gastric cancer progression. Specifically, Wnt5a was shown to upregulate laminin γ2 promoting gastric cancer cell aggressiveness [61]. Moreover, our group has demonstrated that gastric cancer cells with E-cadherin dysfunction depend on laminin γ2 to survive and invade. We postulate that laminin γ2 upregulation may constitute an adaptive stimulus to allow cells to escape anoikis and invade adjacent tissues, contributing to cancer progression [62].

Deregulation of additional ECM components, namely glycoproteins and proteins of the basement membrane, has also been investigated. Lumican, an ECM proteoglycan, was found to be highly expressed in human gastric CAFs and its expression positively associated with depth of invasion, lymph node metastasis, TNM stage, and poor survival rate of gastric cancer patients [63]. Fibulin 1, which belongs to a family of extracellular glycoproteins, is a structural component of the basement membrane able to interact with other ECM components [71]. Importantly, fibulin 1 was shown to be downregulated through promoter hypermethylation in human gastric carcinoma tissues [64]. The levels of Nidogen-2 were reported to be significantly increased in gastric cancer tissues in comparison with normal controls and positively associated with TNM stage and poor prognosis of gastric cancer patients [65]. Connective tissue growth factor (CTGF) is another matrisome glycoprotein with high expression in tumor tissues and found to be an independent predictor of poor prognosis in gastric cancer patients [66,72]. Periostin, a secretory protein that can alter the remodeling of the ECM, was found to be highly expressed in gastric tumors and to be positively associated with gastric cancer metastasis by promoting tumor metastasis and invasion [67,73]. Of relevance, periostin has been reported to maintain primary tumor growth, as well as to contribute to a “fertile soil” for colonization and proliferation of cancer cells in metastatic niches [73,74]. Two different types of ECM proteoglycans, versican, and decorin, were significantly increased in human gastric carcinoma samples when compared with human normal gastric mucosa specimens [68]. Expression of the proteoglycan biglycan was also described to correlate with aggressiveness and poor prognosis of gastric cancer [69]. Interestingly, a core matrisome gene signature, of nine upregulated ECM genes, was identified in patients with gastric, ovarian, lung, and colon cancers, and was able to predict clinical outcome in these patients [75].

Notably, some ECM molecules have been described to be differentially expressed in distinct types of gastric cancer. Indeed, higher expression levels of galectin-1 and thrombospondin were detected in diffuse gastric cancer in comparison with those found in perifocal and tumor zones of the intestinal-type, which could reflect the dissimilarities of the two histotypes [70]. The expression signature of the diffuse gastric cancer included genes encoding collagens, biglycan, osteoglycin, proteoglycan, MMPs, cadherin 11, Thy-1 SERPINS, and fibrillin, revealing active ECM production and remodeling, as well as signaling linked to regulation of cell proliferation [76]. A comparative study of gene expression profiles from diffuse and intestinal-type gastric cancers demonstrated that the signature of the diffuse-type cancer exhibited altered expression of genes related to ECM components, whereas that of the intestinal-type revealed distinct alterations in cell growth or cell cycle pathways [77,78].

**Figure 2:**
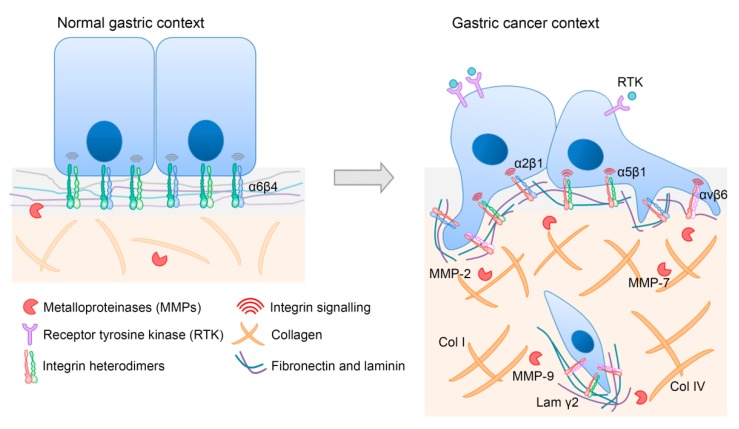
Representative image of major ECM-integrin alterations in gastric cancer. ECM composition, as well as integrin expression and signaling, are distinct in normal and in gastric cancer contexts. Relevant players for gastric carcinogenesis are depicted to illustrate aberrant features. Briefly, during gastric cancer development, the expression of some integrin heterodimers is lost (α6β4), whereas that of others is increased (α2β1, α5β1, and αvβ6). Moreover, the increased activity of several MMPs (MMP-2, MMP-7, and MMP-9) is associated with cancer cell invasion. Increased levels of collagens (Col I and Col IV) and laminin γ2 are also part of a specific gastric cancer ECM signature.

In conclusion, disruption of the tightly orchestrated ECM organization will compromise gastric tissue structure and function, ultimately contributing to gastric cancer progression. In line with this, other key players including integrin receptors that mediate the cell’s interaction with the ECM are crucial determinants for carcinogenesis, as illustrated in Figure 2.

## 4. Aberrant Expression of Integrins in Gastric Cancer

Integrins are cell-surface adhesion molecules that sense information within the ECM and translate such signals into cellular responses involving tissue-specific gene regulation [79]. Remarkably, intracellular signal inputs are also reflected in integrin activity and engagement to ECM ligands, highlighting a bidirectional function of integrins as mechanosensors and mechanotransducers [5].

During outside-in signaling, the engagement of integrins with ECM ligands induces a conformational change on the integrin’s cytoplasmic domain and promotes the assembly of macromolecules termed focal adhesions [80,81]. In contrast, molecular interactions with the integrin cytoplasmic domain lead to conformational changes, resulting in receptor activation and increased affinity to ECM ligands (inside-out signaling) [82,83,84].

Integrins are found as heterodimeric combinations of 18 α and 8 β subunits that interact in a restricted manner, generating 24 family members expressed in a cell- and tissue-specific manner [6]. A combination of both subunits determines integrin specificity for its corresponding ligand. Integrins can recognize Arg-Gly-Asp (RGD) peptide motifs or conformational structures encompassing different amino acid rearrangements [85]. More so, a panel of integrins bind to unique ECM ligands (for instance, α5β1 integrin to fibronectin), whereas others present a certain degree of functional redundancy and are able to engage multiple ligands, overlapping with different integrin heterodimers (such as αvβ3 integrin that binds laminin, collagen, fibronectin, and tenascin C) [86,87]. It has been speculated that this integrin redundancy is an adaptive mechanism to allow a prompt response to changes in the microenvironment [84].

Given the relevance of integrin-ECM interactions in determining cell fate, it is not surprising that deregulated integrin expression and activity is a precursor event in the pathogenesis of many human diseases [88]. Indeed, defects in platelet integrin αIIbβ3 (GPIIb-IIIa) can originate Glanzmann thrombasthenia [89], and the leukocyte adhesion deficiency (LAD) is a primary immunodeficiency disorder caused by a mutation on β2 integrin [90,91]. Additionally, a number of skin diseases are frequently associated with mutations on α2, α6 and β4 integrins [92].

Aberrant integrin expression has also been reported in cancer, where it is associated with progression and poor prognosis [3,6]. In vitro and in vivo studies have demonstrated that abnormal integrin levels award cancer cells with increased capabilities to survive and migrate in a hostile microenvironment [3,6]. Accordingly, over the past years, distinct integrins have emerged as prognostic biomarkers and potential therapeutic targets in the oncology field [3,93,94]. For instance, loss of β1 integrin expression precludes tumor progression in different tumor models, including breast cancer and pancreatic tumor β-cells [95,96]. In early-stage non-small cell lung cancer, increased expression of β1 or α5 integrins was found correlated with poor prognosis [97]. In fact, expression of αvβ3, αvβ5, α5β1, α6β4, α4β1, αvβ6, and αvβ8 integrin heterodimers have been shown to correlate with disease progression and poor patient outcome in a myriad of cancer types and, as such, those have become main targets of research and clinical studies [6].

Focusing on the context of gastric cancer, several integrin heterodimers have already been claimed to be involved in the etiology of the disease (Table 2). In particular, the predictive value of αvβ6 integrin has been highlighted, since its increased expression is recurrently associated with lymph node metastases in gastrointestinal cancers and reduced patient survival [98,99,100,101,102,103]. Zhao et al. reported that the invasiveness of gastric cancer cells expressing αvβ6 integrin seems to occur through ECM degradation in a process mediated by the pro-angiogenic growth factor VEGF and enhanced secretion of matrix metalloprotein-9 (MMP-9) [101]. Another study by Gu and colleagues demonstrated a potential link between cancer cell survival and αvβ6/MMP-9 signaling in colon cancer cells [104]. Additionally, increased αvβ6 expression has been correlated with the number of CAFs, awarding αvβ6 a prognostic value in human gastric cancer [102].

Integrins α2β1 and α3β1 have also been linked to the metastatic process in human gastric cancer, despite exerting separate functions in this process [105,106]. α2β1 was associated with the presence of lymph node and liver metastases, whereas α3β1 expression correlated with liver and peritoneal metastases [105]. Importantly, a multivariate analysis of both integrins in primary gastric cancer samples associates α3β1 expression with peritoneal metastasis formation and depth of invasion [105]. Later, up-regulation of integrin α2β1 was shown to be essential for peritoneal dissemination of gastric cancer promoted by the interaction with Cysteine-rich 61 [106]. Cysteine-rich 61 is an ECM protein regulating a broad range of cellular activities, including cell adhesion, migration, proliferation, cell survival and angiogenesis [109,110]. In breast cancer, Cysteine-rich 61 supports metastases and mitigates anoikis, which may explain its association with more advanced disease features [111,112].

The classical fibronectin receptor, α5β1 integrin, has also been described as abnormally expressed in gastric cancer [107]. A study involving 186 gastric cancer samples demonstrated that 68.3% of cases presented higher α5β1 integrin expression than that of paired normal mucosa [107]. α5β1 integrin levels were closely related to histological differentiation, lymph node metastases, and tumor recurrence, suggesting α5β1-integrin as a marker of poor prognosis [107]. Further, overall survival and disease-free survival of patients displaying high α5β1-integrin expression were significantly worse than those of patients with low or absent expression [107]. In accordance, it was verified that under controlled in vitro conditions, invasive cancer cells display high α5β1 integrin levels [113]. α5β1 generates higher contractile forces and increased cytoskeletal dynamics, which allow faster and persistent migration [113].

More recently, Boger et al. have evaluated αvβ3 and αvβ5 as prognostic, diagnostic and therapeutic targets in a large cohort of 482 gastric cancer cases [108]. The group observed more often a positive αvβ3 and αvβ5 status in the intestinal-type gastric cancer than in the diffuse-type and, thus proposed that both markers could be helpful in the histological classification of gastric cancer [108]. Moreover, αvβ5 was confirmed to be an independent prognostic factor of intestinal-type gastric cancer, given that patients with absence of αvβ5 on stroma cells had better disease outcome and significantly longer survival [108]. In an in vivo context, blockage of αvβ3 and αvβ5 integrins with monoclonal antibodies resulted in a drastic reduction of tumor growth and metastases through inhibition of focal adhesions and cell motility signals [114,115]. Notably, loss of integrin expression has also been described in gastric cancer. A study by Ishii et al. demonstrates that loss of α6β4 is a biomarker of peritoneal dissemination and poor prognosis of gastric cancer patients [116]. Integrins play an indisputable role in nearly every step of cancer progression, from initiation to metastasis [6]. In gastric cancer, it is also clear that altered integrins mediate an array of cellular effects that culminate in tumor progression. An outstanding challenge in this research field remains the understanding of the signaling following integrin-ECM gastric interactions that trigger tumor-promoting characteristics.

## 5. ECM-Integrin Signaling in Cancer

Every single cell type displays a specific integrin expression profile that changes in response to cellular or environmental inputs [117,118]. In normal cells, integrin activity is strictly regulated, whereas in cancer cells, abnormal integrin activity promotes the acquisition of oncogenic properties either through ECM remodeling or by interfering with intracellular signaling that may, for instance, lead to oncogene activation [6,117]. Tumor cells are able to switch integrin’s exposure and modify their downstream signaling in order to survive, proliferate and successfully colonize adjacent tissues [119]. In breast cancer, it has been reported that decreased levels of α2β1 and α3β1 integrins potentiate tumor cell dissemination [95,120,121]. In contrast, overexpression of α6β4 and αvβ3 integrins were shown to be correlated with metastasis formation and shorter patient survival [122,123,124]. The processes underlying integrin regulation and signaling in cancer are however complex and highly dependent on the tissue of origin, histological tumor type, and disease stage [119].

As explained in the previous section, integrins hold a unique ability to signal bidirectionally and can thus initiate a cascade of events upon either ligand engagement or intracellular interaction with specific moieties [125,126]. Integrin binding to ECM proteins induces integrin clustering and assembly of focal adhesion complexes at the plasma membrane. The tyrosine-phosphorylated protein Focal Adhesion Kinase (FAK) is a major component of focal adhesions that, upon recruitment, is autophosphorylated at Tyr397 residue, exposing a steroid receptor coactivator (Src) homology 2 (SH2) domain-binding site for Src [127,128]. Src then phosphorylates FAK at the additional Tyr576 and Tyr577 residues, amplifying its catalytic activity [92,129]. FAK phosphorylation mobilizes proteins that contain SH2 domains, such as the growth factor receptor-bound protein 2 (Grb2) and the phosphatidylinositol 3-kinase (PI3K), which in turn activate the Ras-mitogen-activated protein kinase (MAPK/ERK) and PI3K pathways [130,131]. In particular, binding of α5β1 to fibronectin, or binding of the epidermal growth factor (EGF) to its receptor (EGFR), triggers MAPK/ERK signaling that coordinates cell survival, proliferation, motility and metabolism [6,132,133,134]. The PI3K/AKT pathway is preferentially activated in response to αvβ3 integrin, which promotes survival of cancer cells by targeting the pro-apoptotic Bcl-2 related protein [135]. Under conditions of nutrient availability, activation of PI3K/AKT can also involve the mammalian target of rapamycin (mTOR) that controls cell growth and proliferation [136].

Additionally, activated FAK can bind and phosphorylate other molecules, namely paxillin and p130Cas (also known as BCAR1), allowing the subsequent recruitment of a panel of adaptor and signaling molecules [127,137]. Through recruitment and phosphorylation of p130Cas, activated FAK/Src complex stimulates Rac1 activity, a member of the Ras superfamily of GTP-binding proteins that induces protrusion formation and inhibits cytoskeletal contractility, thereby facilitating cell spreading [138].

Corroborating the involvement of integrin-mediated signaling in the genesis and development of gastric cancer, pTyr397 FAK phosphorylation was found to be abundant among patients with gastric carcinomas [139]. Patients with higher levels of pTyr397 FAK displayed increased recurrence following surgical resection and poor 5-year recurrence-free survival [139]. In vitro studies demonstrated that a dominant-negative mutant of FAK (impairing the Tyr397 FAK phosphorylation) reduced the ability of gastric cancer cells to migrate, invade, and proliferate when compared with cells overexpressing wild-type FAK [139]. The pathogen *H. pylori* has also been shown to activate FAK in gastric epithelial cells, leading to cell scattering and elongation [140]. Upon translocation of the bacterial factor cytotoxin-associated gene A (CagA), FAK activity is modulated by both cortactin and vinculin modifications, which deregulate cell-matrix adhesion [140,141]. Moreover, expression of p130Cas was mainly absent in normal gastric mucosa, whereas it was strongly or moderately positive in gastric carcinoma [142]. A similar tendency was observed for paxillin, which was aberrantly upregulated in gastric cancer tissues and cell lines [143,144]. In fact, Chen and collaborators evaluated a large series of 239 gastric cancer patients and established a direct correlation between paxillin expression and distant metastasis, as well as advanced tumor stage [143]. Protein modulation through overexpression and inhibition approaches revealed that paxillin is a key regulator of proliferation and migration of gastric cancer cells [143].

In contrast with the outside-in cascade of events, inside-out signaling initiates upon binding of integrin-activators like talins and kindlins (kindlin-1, kindlin-2, and kindlin-3) to the intracellular portion of β-integrins [92,145]. This interaction leads to an extended conformation of integrins and, consequently, to their increased affinity for ECM ligands [92,145]. Remarkably, kindlin-2 was upregulated both at RNA and protein levels in gastric cancer [146]. High kindlin-2 expression levels were associated with tumor stromal invasion, lymph node metastasis, and tumor staging, and were considered an independent risk factor of progression-free survival [146]. In this context, kindlin-2 seems to play a pro-invasive function through the activation of β1 and β3 integrins [147].

Aside from its function as an integrin activator, talin is also a critical mediator of mechanotransduction signals [148]. Along with filamin and α-actinin, talin is responsible for the connection between integrins and the actomyosin cytoskeleton [149]. This cytoskeletal bridge is crucial to orchestrate protein trafficking, cell morphology and a myriad of cellular functions, including survival and motility [14]. Unlike talin, kindlins alone are not sufficient to shift integrins to a high-affinity state, despite being required for proper talin function [150]. The mechanism through which kindlins cooperate with talin to support integrin activation remains unclear, although it has been proposed that kindlins recruit talin to integrin β tails, promoting integrin activation [151]. A different explanation is that kindlins and talin synergize in integrin activation and do not interfere with each other´s interaction with integrins [152]. Accordingly, kindlins may co-activate integrin through a mechanism independent of talin recruitment [152].

Despite the increased knowledge of the signaling cascades mediating cell-ECM interactions, there is still a lack of studies focusing on gastric cancer. In the near future, we expect to witness breakthrough research in this topic unraveling disease-associated mechanisms and, ultimately, fostering the emergence of novel therapeutic strategies targeting integrin signaling.

## 6. Potential Therapeutic Targets and Strategies

Several studies have shown that inhibition of integrin or its downstream effectors could block the major hallmarks of cancer [3,119]. Therefore, integrins and adaptor molecules have soon emerged as potential therapeutic targets for a number of cancer types, including glioblastoma, melanoma and breast cancer [115,153,154,155,156].

Based on integrin expression profiles, two therapeutic strategies have been developed. One involves direct inhibition of integrin function and the other aims at integrin-directed delivery of drugs, with the first concept being employed more often in the clinic, namely in ulcerative colitis, Crohn’s disease, and multiple sclerosis [3,157].

So far, no clinical trials of integrin-based therapies have been carried out for gastric cancer (ClinicalTrials.gov). This is probably due to scarce data regarding the integrin expression profile in gastric carcinoma patients and in normal gastric tissue.

Among the few ECM receptors described as abnormally expressed in gastric cancer, ανβ6 increased expression is associated with reduced survival and it has been suggested as a prognostic marker in early-stage disease [99,100]. As such, ανβ6 could be an attractive target for early intervention and treatment of gastric carcinoma, and to date, several antibodies and small molecules have been developed to inhibit this molecule. Abituzumab (DI17E6, EMD 525797: Merck KgaA)—a humanized monoclonal IgG2 antibody that targets αν heterodimers [158] – yielded clinical benefit in patients with early-stage metastatic colorectal cancer expressing high levels of ανβ6 [159]. Intetumumab (CNTO95) from Centocor is also a pan-αv integrin inhibitor with well-established anti-tumor and anti-angiogenic effects in a human melanoma xenograft model [114,115]. In Phase I trials, it exhibited low toxicity and good tolerance among patients affected by advanced melanoma and castration-resistant prostate cancer [160,161]. Still, the therapeutic effect of Intetumumab in both cancer contexts requires further investigation [160,161]. More recently, Biogen-Idec developed a monoclonal antibody specifically targeting ανβ6, which was shown to inhibit tumor growth in xenografts of human pharyngeal carcinoma cells through regulation of transforming growth factor-beta (TGF-β) [162]. This antibody, STX-100 (BG00011), is currently in clinical trials for treatment of idiopathic pulmonary fibrosis and for nephropathy [157]. Likewise, the first small molecule inhibitor of αvβ6 integrin, GSK3008348, was produced by GlaxoSmithKline Research as an inhaled compound for the treatment of idiopathic pulmonary fibrosis [163].

As described in previous sections, α2β1 integrin was found overexpressed in peritoneal metastases of gastric carcinoma and has been implicated in the dissemination of gastric cancer cells, both in patient samples and xenograft models [164,165]. Aside from its role as a regulator of cancer metastasis, α2β1 was described as a promoter of inflammation, angiogenesis, and chemoresistance [166]. Hence, several clinical programs have been implemented targeting this specific molecule. For instance, the monoclonal blocking antibody Vatelizumab (CHR-1103) was developed by Chromos Molecular systems and Glenmark Pharmaceuticals for the treatment of multiple sclerosis and ulcerative colitis [157,166]. The sulfonamide derivative small molecule E7820, which inhibits α2 gene expression, was used in combination with standard chemotherapy in advanced or refractory solid tumors, namely metastatic colorectal carcinoma [167,168].

α3β1 integrin is a receptor of laminin and its expression is correlated with the depth of gastric cancer invasion (into the muscularis propria or subserosa), as well as with the formation of peritoneal metastases [105]. Interestingly, an existing pan-specific anti-β1 antibody targeting laminin receptors was shown to exert a synergistic anti-tumor effect when combined with cisplatin [169].

Despite encouraging results from in vitro and preclinical studies, the success of integrin-targeted strategies in cancer has been limited [157]. In fact, the applicability of ECM receptors as therapeutic targets is highly dependent on the tumor type and on the disease stage given that the pattern of integrin expression varies between cancer types and during cancer progression [6]. Careful patient stratification and a deep understanding of basic mechanisms of integrin regulation are thus urgent to improve the anti-tumor efficacy of integrin therapies.

In addition to integrin-targeted strategies, ECM remodeling may also be beneficial for cancer treatment. Several cancer types exhibit abnormal accumulation/deposition of particular ECM components and increased ECM stiffness, which impair drug diffusion and, consequently, decreases treatment efficacy [4,170]. Moreover, an increased ECM density perturbs cell-cell adhesion, enhances cell-ECM interaction and increases the proliferation of gastric cancer cells [60]. Therefore, modulation of ECM-related enzymes such as collagenase, MMPs or lysyl oxidases can be a promising therapeutic strategy for gastric cancer. Collagenase disrupts collagen networks and stimulates anti-tumor immune surveillance by increasing the ability of T cells to interact with lung cancer cells [171]. MMP inhibitors, such as Marimastat, Batimastat, and Prinomastat, failed all trials in various cancer types due to the broad-spectrum of these drugs (acting simultaneously in anti- and protumorigenic MMPs) [172,173,174,175]. However, individual MMP-targeting approaches using monoclonal antibodies have been gaining attention and will certainly improve their tolerability and efficacy [176]. In contrast to collagenases and MMPs, which are ECM degrading enzymes, lysyl oxidases generate covalent cross-links between collagen fibers, inducing tissue stiffness and ECM resistance/stability [177,178]. The administration of a monoclonal antibody inhibiting lysyl oxidase-like 2 (LOXL2) reduced lung and liver fibrosis, as well as metastases in xenografted tumors [179]. Those effects were associated with a decrease in activated fibroblasts, reduced production of growth factors and cytokines, and inhibition of TGF-β signaling [179].

Previous studies have demonstrated that TGF-β is increased in gastric tumor tissue when compared with adjacent mucosa [180]. In addition, high TGF-β expression was correlated with worse overall survival of gastric cancer patients [180]. Given the importance of TGF-β in profibrotic activity and in the regulation of ECM synthesis, secretion, and processing [181,182], several studies were designed to evaluate the response of TGF-β inhibitory antibodies [183,184,185]. A specific monoclonal antibody, Fresolimumab (GC1008), was tested in advanced melanoma and renal cell carcinoma patients with acceptable safety and evidence of anti-tumor activity [183]. In systemic sclerosis, Fresolimumab was shown to decrease dermal myofibroblast infiltration and to reduce expression of fibrosis markers [184].

The angiogenic process is also known to involve interactions between endothelial cells and the ECM. Intensive research focusing on this critical interaction has unveiled anti-angiogenic factors with application in cancer therapeutics, namely small peptides derived from naturally occurring proteins. Endostatin is a small fragment of the ECM protein collagen type-XVIII identified as a potent inhibitor of angiogenesis [186]. In gastric cancer, patients displayed higher serum endostatin levels than those of healthy subjects, which were correlated with aggressiveness [187]. Recombinant human endostatin, Endostar, proved to be more effective than single chemotherapy in a plethora of cancers, including gastric cancer [188,189]. Additionally, various sites for angiogenesis have been identified on laminin-1, among which, C16Y was shown to be a potent antagonist to integrins during angiogenesis and has thus been suggested as a potential cancer therapeutic agent [190].

One different anti-angiogenic strategy encompasses therapies targeting the angiogenic VEGF-mediated pathway, which is considered critical not only for the regulation of tumor angiogenesis but also for the degradation and remodeling of the ECM [191]. Significant evidence awards VEGF/VEGFR2 signaling an important role in gastric cancer pathogenesis, and indeed, gastric cancer patients were reported to display significantly higher plasma or serum VEGF levels than healthy control subjects [192]. The monoclonal anti-VEGF antibody Bevacizumab, which was the first drug targeting the VEGF pathway, did not reach promising results in overall survival of gastric cancer patients in the AVAGAST clinical trial, however, it is now approved for first- and/or second-line treatment of a variety of tumors including colorectal cancer [191]. In contrast, the human monoclonal anti-VEGFR2 antibody Ramucirumab yielded significant survival benefits in patients with previously treated advanced gastric cancer or gastroesophageal junction carcinomas in a phase III clinical trial [193]. Accordingly, Ramucirumab is now used to treat gastric cancer patients with advanced or metastatic disease on or after first-line chemotherapy [191]. Currently, there are also a number of clinical trials addressing the inclusion of Apatinib, a highly selective VEGFR2 inhibitor, in gastric cancer treatment regimens, including phase III and IV trials [191,194].

It is clear from the extensive research and clinical trials that despite the enormous potential in modulating the ECM, the majority of agents elicit therapeutic responses in gastric cancer patients that are often too modest. Given that the ECM is actively remodeled, a major challenge in drug development is to identify predictive biomarkers and the correct therapy timing, as well as to design clinical trials with enriched populations, so gastric cancer patients can be offered treatments that will result in a significant increase in overall survival.

## 7. Conclusions

Increasing evidence has shown that a specific ECM signature is associated with each tissue and its functional features. ECM dynamics, composition, and structure are tightly regulated, and ECM remodeling has proven to have a major impact on cancer progression and prognosis. Experimental and clinical observations strongly indicate that ECM composition and cell exposure of specific integrins are accomplices in the precancerous cascade leading to gastric cancer, namely by promoting proliferation, survival, migration, invasion, and metastasis. Therefore, ECM constituents, receptors and associated signaling molecules should be explored as biomarkers of prognosis and/or therapeutic targets. Innovative and more effective gastric cancer treatments can be achieved by using combined strategies of ECM targeting with RTK inhibitors or immuno-oncology agents.

## Figures and Tables

**Table 1:** ECM components deregulated in gastric cancer. ECM proteins displaying abnormal expression patterns in gastric cancer and associated clinical observations.

ECM Component	Clinical Relevance and Pathological Findings	References
Tenascin	Increased expression in pre-malignant and malignant gastric epithelia (diffuse and intestinal types).	[7]
MMP-2	Increased production in the gastric mucosa of patients with *H. pylori*-associated gastritis;	[46]
	Higher expression in intestinal-type than diffuse-type gastric cancer;	[49]
	Higher expression associated with poor prognosis.	[50,51]
MMP-7	Higher expression associated with aggressive tumor phenotype and shorter overall survival.	[52]
MMP-9	Increased production in the gastric mucosa of patients with *H. pylori*-associated gastritis;	[46]
	Increased expression associated with depth of cancer invasion;	[53]
	Increased levels in serum of gastric cancer patients;	[54]
	Increased expression in GIST;	[55]
	Higher expression in intestinal-type than diffuse-type gastric cancer;	[49]
	Higher expression associated with poor patient prognosis.	[56]
MMP-9/NGAL	Higher levels in urine of gastric cancer patients.	[57]
COL12A1	Overexpression correlated with tumor invasiveness, metastasis, and advanced clinical stage.	[8]
COL1A1	Overexpression correlated with overall survival;	[9]
	Differentially expressed in pre-malignant and malignant lesions of the human stomach.	[10]
COL4A1	Overexpression correlated with overall survival.	[9]
COL11A1	Differentially expressed in pre-malignant and malignant lesions of the human stomach.	[10]
COL6A3	Overexpressed in gastric cancer tissues.	[58]
Collagen	Deregulated collagen metabolism.	[59]
Collagen I	Higher levels in tumor tissues.	[60]
Collagen IV	Higher levels in tumor tissues.	[60]
Fibronectin	Higher levels in tumor tissues.	[60]
Laminin	Higher levels in tumor tissues.	[60]
Laminin γ2	Mediates Wnt5a-induced invasion of gastric cancer cells;	[61]
	Upregulated in gastric cancer and involved in cancer progression.	[62]
Lumican	Expression associated with depth of invasion, lymph node metastasis, TNM stage, and poor survival rate.	[63]
Fibulin-1	Downregulated through promoter hypermethylation.	[64]
Nidogen-2	Overexpressed and associated with the TNM stage.	[65]
CTGF	Predictor of poor prognosis.	[66]
Periostin	Higher expression associated with metastasis.	[67]
Versican	Increased in gastric cancer samples.	[68]
Decorin	Increased in gastric cancer samples.	[68]
Biglycan	Expression correlates with aggressiveness and poor patient prognosis.	[69]
Galectin-1	Higher expression in diffuse-type than intestinal-type gastric cancer.	[70]
Thrombospondin	Higher expression in diffuse-type than intestinal-type gastric cancer.	[70]

**Table 2:** Integrins abnormally expressed in gastric cancer. Depiction of reported integrins with aberrant expression pattern in gastric cancer and their clinical relevance.

Integrin	Clinical Relevance and Pathological Findings	References
αvβ6	Positive expression is linked to significantly reduced survival;	[100]
	Induces invasion through ECM degradation in a process mediated by VEGF and MMP-9;	[99,101]
	Increased αvβ6 expression correlated significantly with the number of CAFs, awarding αvβ6 a prognostic value in human gastric cancer.	[102]
α2β1	Correlated with the presence of lymph node and liver metastases;	[105]
	Essential for peritoneal dissemination of gastric cancer promoted by Cysteine-rich 61.	[106]
α3β1	Independent factor associated with increased liver and peritoneal metastases; Correlated with the depth of invasion.	[105]
α5β1	Increased expression in gastric cancer patients associated with histological differentiation, lymph node metastases, and tumor recurrence; Proposed marker of poor prognosis.	[107]
αvβ3	Positivity correlates with intestinal-type gastric cancer.	[108]
αvβ5	Positivity correlates with intestinal-type gastric cancer; Independent prognostic factor of poor patient outcome.	[108]
